# Policy and Programming Towards Addressing Treatment Gaps in Adolescents Living with HIV: A Content Analysis of Policy and Programme Documents in Namibia

**DOI:** 10.1177/23259582241236061

**Published:** 2024-03-06

**Authors:** Farai K. Munyayi, Brian E. van Wyk

**Affiliations:** School of Public Health, 56390 University of the Western Cape, Cape Town, South Africa

**Keywords:** HIV, antiretroviral treatment, policy, adolescents, viral suppression

## Abstract

Adolescents living with HIV (ALHIV) face unique challenges resulting in persistent treatment gaps, particularly viral non-suppression. Country programs adopt policies, guidelines, and innovations, based on WHO recommendations and best practices from elsewhere. However, it is unclear to what extent these tools address the management of adolescents with viral non-suppression. We report on a review of guidelines for the provision of HIV services to ALHIV in Namibia. We conducted a systematic document review using Content Analysis and Thematic Analysis methodology, and the READ approach. We identified seven relevant policy documents, four of which somewhat addressed viral non-suppression (treatment gap) in ALHIV and outlined interventions to improve treatment outcomes in adolescents considering their lived experience and unique challenges. The persistent treatment gap may reflect policy implementation gaps in specifically addressing viral non-suppression. It may be worthwhile to leverage existing documents to develop specific operational guidance for ALHIV with unsuppressed viral loads.

## Plain Language Summary

Study analysing the potential gaps in the contents of policies and programme documents meant to address management of adolescents living with HIV with high viral load

Viral load suppression is a huge challenge in adolescents living with HIV (ALHIV). Globally, adolescents lag when compared to children and adults in achieving viral suppression levels set for achieving HIV epidemic control. The WHO and global HIV program initiatives recommend evidence-based interventions to be included in policies and guidelines to address unique barriers adolescents face that prevent them from staying in HIV care and adhering to their medication. The extent to which country policies guide service providers in managing high viral load cases among adolescents is important in identifying and addressing the persistent gaps. We reviewed the contents of policies, guidelines and other programmatic documents that address HIV management in adolescents in Namibia to assess the extent to which the documents guide management of ALHIV who have high viral load. Seven documents addressing management of ALHIV in Namibia were identified. Four documents address viral suppression among adolescents and recommend some interventions to improve treatment outcomes in adolescents in general. The documents acknowledge the uniqueness of the adolescence, with unique experiences and challenges. However, the documents fall short in providing comprehensive and specific guidance in managing adolescents with high viral loads, for program implementers and direct service providers for ALHIV. The fragmented guidance on managing adolescents with unsuppressed viral loads may be leading to implementation gaps or uncertainties among service providers on how to manage unique cases. It would be essential to focus future efforts on consolidation or development of comprehensive guidance on management of adolescents with high viral load, and capacitating the healthcare providers and stakeholders engaged in addressing social determinants of health affecting these adolescents. A multisectoral approach may provide a pathway to improved viral suppression among ALHIV.

## Introduction

Global efforts to achieve HIV epidemic control include commitments by national HIV programs to develop and implement strategies to meet the revised UNAIDS 95-95-95 targets, which aim to ensure that 95% of all PLHIV know their status, 95% of all HIV positive are initiated and retained on antiretroviral therapy (ART), and 95% of all individuals on ART achieving viral suppression.^1^ The WHO has issued a series of normative guidance documents on the implementation of ART programs across the globe, to optimize national HIV treatment programs and achieve maximum gains using evidence-based interventions. In addition, global funding mechanisms for the HIV response, such as the US President’s Emergency Fund for AIDS Relief (PEPFAR), the Global Fund for AIDS, TB and Malaria, and several others, continue to issue updated guidance for effective and efficient HIV response programming, in an era of dwindling resources.

Considering the resources that multinational organizations and country programs have committed to the HIV response, many countries are close to achieving the 95-95-95 goals, while others have achieved the targets and hence are classified as countries that have reached epidemic control. However, the gains made in achieving the 95-95-95 goals remain elusive in other sub-populations, especially in children, adolescents and youth living with HIV (CAYLHIV). Globally, by 2022, 63% [49–86%] of children living with HIV (CLHIV) knew their HIV status, 57% [44–78%] were receiving ART (91% of children who knew their status), and 46% [36–63%] of CLHIV had a suppressed viral load (representing 81% of children on ART).^2^ However, availability of disaggregated data for adolescents in sub-Saharan Africa is limited although understanding of the state of the 95-95-95 targets among this sub-population, especially viral suppression, is essential for policymakers for tailoring interventions targeting adolescents.^3^ A multiregional retrospective cohort study conducted in 31 countries reported that, adults living with HIV are approaching the 95% target for viral suppression, while CALHIV still lag behind with viral suppression levels of 59% 3 years post ART initiation.^4^

The 2020 global HIV data estimates showed that about 1.75 million [1.16 million–2.3 million] adolescents were living with HIV, reflecting an increasing population of adolescents living with HIV (ALHIV) globally as compared to the previous years.^5^ The WHO describes adolescence as the life phase following childhood, which precedes reaching adulthood, strictly ages 10 to 19 years old.^6^ The estimates from 2020 alone showed that there were approximately 150,000 new HIV infections among adolescents worldwide.^5^ The persistently high number of new HIV infections among adolescents and a considerable number of vertically-infected children who are surviving into adolescence due to advances in HIV treatment, adds to the growing sub-population of ALHIV.^7^ Achieving the aspired levels of treatment outcomes in this growing sub-population of ALHIV remains a great concern as they face unique barriers and challenges to access to HIV services, and continue to be underserved by most health systems, especially in sub-Saharan Africa. The HIV services in many sub-Saharan African countries are organized around paediatric HIV care and adult care and adolescents often fall and are lost in the transition between paediatric and adult care. Often, the specific needs of adolescents (10-19 years) are masked by existing policies that address paediatric issues (0-14) and adults (15 + years).^8,9^ Policies then fall short in addressing adolescent specific challenges and promoting new innovations such as harnessing digital technologies to reach adolescents or improve adherence in challenging settings for adolescents such as boarding schools.^2^

In most countries in sub-Saharan Africa, the public healthcare facilities are mostly ill-equipped to provide adequate support and guidance for ALHIV to stay engaged in care and achieve viral suppression as the HIV services are designed to serve the younger children in pediatric HIV clinics or the adult clients in adult care clinics.^8^ While CAYLHIV continue to be underserved by many healthcare systems regarding access to appropriate and adequate HIV services, the plight of adolescents was exacerbated by the COVID-19 pandemic in recent years, with implementation of restrictive measures to mitigate the spread of SARS-CoV-2 inadvertently affecting access to health facilities.^10^ If the current trajectory continues unabated, not only would the goal to achieve epidemic control remain a far-to-reach goal, but the significant progress towards epidemic control made during the pre-COVID-19 period may be reversed. Increased focused and concentrated efforts are needed on ALHIV to get back on track and make progress towards achieving HIV epidemic control.^5^ More accelerated and focused efforts targeting adolescents and young people living with HIV (AYLHIV) are essential to make significant progress towards achieving overall HIV epidemic control.^5^

In light of the apparent challenges with ALHIV and the need to close the treatment gap among this sub-population, the global HIV response and country HIV programs have developed a number of policy guidance, initiatives and programming tools that are focused on ALHIV. In 2019, the WHO in collaboration with the Elizabeth Glaser Pediatric AIDS Foundation (EGPAF) and other partners, issued the “AIDS Free Framework to accelerate paediatric and adolescent HIV treatment”, which was a collaborative framework for accelerating the end of the AIDS epidemic among children, adolescents and young women by 2020.^11^ The collaborative framework circumscribes to the 2016 Political Declaration on HIV and AIDS goals set by the United Nations Member States which stated that every CALHIV should have access to ART and builds on the successes of the “Global Plan towards ending new HIV infections among children by 2015 and keeping their mothers alive”.^11^

The UNICEF, UNAIDS and other international partners launched the “ALL in! to End Adolescent AIDS”, in 2015, a global initiative with targets for 2020 to end AIDS among adolescents by 2030.^5^ Efforts to address the diverse and unique needs of ALHIV to improve their treatment outcomes needs an integrated and comprehensive approach. Such efforts rely on global approaches and commitments to adolescent health, such as the “Global Strategy for Women's, Children's and Adolescents’ Health (2016-2030)” and the Global Accelerated Action for the Health of Adolescents (AA-HA!).^12^ The WHO, in its technical brief of 2019 provided some recommendations and key considerations for provision of adolescent-friendly HIV services as well as examples of best practices for peer-led interventions for ALHIV.^13^ In addition to other programmatic intervention, the WHO recommends a participatory approach in adolescents’ own care, from planning, implementing, and monitoring and evaluation, implementation of peer-driven programs, with integrated services provision, including psychosocial interventions, to ensure improved treatment outcomes among ALHIV.^12^

Namibiàs HIV epidemic trajectory is similar to other low- and middle-income countries (LMICs). The 2017 Namibia Population-based HIV Impact Assessment (NAMPHIA) study showed that Namibia had attained an estimated 86%-96%-91% towards the 90-90-90 UNAIDS targets, with a reported overall 4.0% HIV prevalence among young people who are aged 15–24 years, and with a 1.7% prevalence of HIV among young adolescents who are aged 10 to 14 years.^14^ Recent spectrum modelling estimates revealed that Namibia has achieved 92% −99%−94% of the revised UNAIDS 95-95-95 targets. However, the overall estimates reported regrettably conceal the gaps that might be existing within distinct sub-populations. Routine program data to categorically monitor performance indicators and HIV treatment outcomes among ALHIV (10-19 years) is often unavailable owing to disaggregation of routinely reported data by <15 years for children and ≥15 years as adults.^15,16^

However, recent studies conducted in Windhoek reported lower retention in care rates and viral suppression in ALHIV compared to adults of 84.6% (at 36 months) and 88%, respectively.^17,18^ To address the unique challenges faced by ALHIV, Namibia has adopted policies, developed guidelines, adopted some innovations and strategies, based on WHO recommendations and evidence from elsewhere, as well as embraced global initiatives specifically addressing adolescent HIV services. However, it is not clear to what extent these policies address the management of ALHIV with unsuppressed viral loads. In this paper, we report on a review of the health policies and guidelines for the provision of HIV care and treatment services to ALHIV.

## Methods

### Methodological Approach

Healthcare policy may be defined as plans, decisions, and actions developed and implemented to achieve specified goals in healthcare provision within a population or society. These plans explicitly define the vision, outline priorities and anticipated roles of individuals or groups, inform the population and build consensus on goals.^19^ Examination and analysis of policy documents can be a powerful method in health policy research, to determine the extent to which the available policy guidance addresses and meets the population healthcare needs.^20^ The READ approach is an example of a systematic approach for conducting a policy document analysis (also known as a documents review) which involves four steps, namely (1) ready your materials, (2) extract the data, (3) analyse your data and (4) distilling the findings.^21^ This approach provides a practical guide to rigorous policy documents analysis to enrich understanding of health policy documents content and processes and discourse around the policies.^21^ Utilizing content analysis (CA) and thematic analysis (TA) methodology, which are both iterative processes that rely on intimate knowledge of the data being studied, provide insights and advanced understanding of the health policies contents and key themes embedded in the policies.^16^ Thematic analysis is an intuitive qualitative research approach that explores patterns across datasets, which entails identifying, analysing and interpreting and reporting repeated patterns in meaning (or themes) in the data.^22^ Themes can be defined as “overarching categories of common information” which may be derived from coded information, which narrates a story regarding dimensions of a phenomenon of interest.^23^

On the other hand, content analysis can be described as a systematic examination of texts to inductively or deductively determine the presence of particular words, concepts, themes, patterns or intent within qualitative data.^24^ This practical approach can be used to explain a certain phenomenon using quantitative or qualitative methods to analyse textual or visual data. The approach facilitates the describing of the characteristics of the contents of the documents by examining what the document says, developed by whom, who it targets, and for what intention and effect.^16^ Both thematic analysis and content analysis serve identical aims of analytically interrogating narrative materials developed for specified intents and purposes “by breaking the text into relatively small units of content and submitting them to descriptive treatment.”^25^

### Data Collection

Considering that our focus was on analysing the content in policy and program documents developed, adopted, or adapted from elsewhere, and under the custodianship of the national government, we iteratively searched the internet for relevant Namibia specific documents made available online in the past 15 years, ie, from 2008 to 2023, that addressed management of ALHIV. We also specifically searched for MoHSS policy and program documents, as the lead ministry with the mandate and carrying the technical responsibility for developing policy for adolescent health, and the overall healthcare services in Namibia. Search terms used included combinations of words such as “adolescent”, “young”, “youth”, “HIV”, “viral load”, “health”, “antiretroviral treatment”, “ART”, “policy development”, “policy”. The latest versions of the documents were considered for inclusion in the final analysis. We also searched for physical copies of relevant documents at the MoHSS, consulted key experts in the field and other stakeholders engaged with interventions to improve treatment outcomes in ALHIV, to ensure that our search was comprehensive enough to include all relevant policy and program documents. The relevance and scope of the documents related to their content addressing management of ALHIV were reviewed by the first and second authors for inclusion in the final analysis.

### Data Analysis

Selected policy and program documents were collated and exported into the Mendeley Reference Manager Software which was used to manage the versions of policy documents and the citations thereof.^26^ A policy analysis framework was developed to capture the characteristics and key contents of each policy and program document that was selected for inclusion in the final analysis. The policy analysis framework was developed to capture the key document identifiers and essential concepts related to management of ALHIV derived from the research questions. Initially, a deductive analytical approach was utilized, organising data using pre-defined terms of interest. The pre-defined data outlined in the documents and extracted included the policy or program document title, year, participants/contributors/stakeholders, purpose of the document, the definition of adolescents, and key content addressing management of ALHIV. Secondly, the scope and relevance of the policy and program documents were reviewed using the presence or absence of key pre-defined terms such as:

- does the document focus on adolescents (10-19 years)?
- does it address viral non-suppression among ALHIV?
- does the document present any interventions to improve treatment outcomes? and
- does the document consider and build on adolescents’ lived experiences?

Utilizing an inductive coding approach, an initial coding framework was then applied to the extracted content from two documents and was reviewed by the first and second author before proceeding to complete the coding process. The codes emerging from the contents were grouped into code categories and further synthesized into emerging themes during the analysis process.

### Ethics Approval

The ethical approval was obtained from the relevant research institution (ref. no. BM21/5/7) and ministry of health (ref. no. 17/3/3/FKM). This study did not involve human subjects nor patient participants therefore, it did not require obtaining verbal or written informed consent.

## Findings

We identified seven policy documents that relate to health service delivery to ALHIV in Namibia, as outlined in Table 1. All seven documents were quite recent – with six released in 2019, and one in 2017.^27–33^ Most (6 out of 7) documents were developed by the Namibia Ministry of Health and Social Services (MoHSS), with the Directorate of Special Programs (DSP) taking the leading role. All documents reflect a collaborative process of policy making which included sectoral and other line ministries, Civil Society Organizations (CSOs), representatives and Implementation Subject Matter Experts (ISMEs) from international development partners such as WHO, UNAIDS, UNICEF, PEPFAR, Global Fund on AIDS, TB and Malaria, NGOs, Healthcare Workers, and most importantly, the adolescents and youth living with HIV and their caregivers. One document, developed by the Zvandiri (As I am) group in Zimbabwe, outlines the Community Adolescent Treatment Supporters (CATS) program, that has been adopted by the Namibia MoHSS, and named the Namibian Adolescent Treatment Supporter (NATS) program.

**Table 1.** Characteristics and Summary of National Health Policies for Management of Adolescents Living with HIV in Namibia.

#	Policy/Document	Year	Participants	Purpose	Adolescent Definition	Content Summary
1.	National Guidelines on Adolescents Living with HIV, second Edition	2019	MoHSS UNICEF Sectoral Ministries, Civil Society Organizations, Development partners ALHIV/Guardians/ Caregivers (formative research)	Framework to guide programmers and service providers in the provision of quality of life and care for ALHIV in Namibia. A minimum basic essential package of care, actions and standards for multisectoral responses that address the holistic needs of ALHIV.	Age: 10–19 years (WHO) Further classified as early adolescence (10-14) and late adolescence (15-19).	**Psychosocial support** Mental health, Counselling, Peer support, HIV disclosure, Caregiver support, Stigma and discrimination, Substance abuse Biomedical interventions HIV treatment, Viral load monitoring, Medication challenges, Switching ART regimens **Structural interventions** Academic life, Schooling, Community support systems, Family structural challenges support, Economic/ financial support, Life skills/Livelihood support, Rights-based universal access to HIV services **Health Services delivery models** Differentiated care service delivery, Adolescent and youth-friendly HIV services, Integrated Healthcare services package for ALHIV, Case management approach, Health Systems optimization, **Multisectoral interventions** Multisectoral collaboration **Independent management of overall health** Self-efficacy, treatment literacy, Transition to adult care, Participatory approach
2.	National Paediatric and Adolescent HIV Care and Treatment Strategy	2019–2023	MoHSS Development partners (PEPFAR, GFATM, NGOs, CSOs, WHO, UNAIDS) AYPLHIV in Teen Clubs	To address the unique challenges in HIV diagnosis, linkage to HIV care, retention in care, adherence to treatment and viral load suppression among adolescents living with HIV.	Age: 0–19 years (Children and Adolescents living with HIV – CALHIV) Recommends finer disaggregation by sex and by smaller age bands to better inform the HIV response in CALHIV (<1, 1-4, 4-9, 10-14 and 15-19 years)	**Psychosocial support** Mental health, Counselling, Peer support, HIV disclosure, Caregiver support, Stigma and discrimination **Biomedical interventions** HIV treatment, Viral load monitoring, Switching ART regimens **Structural interventions** Schooling, Community support systems, Family structural challenges support, Economic/ financial support, Life skills/Livelihood support, Rights-based universal access to HIV services **Health Services delivery models** Differentiated care service delivery, Adolescent and youth-friendly HIV services, Integrated Healthcare services package for ALHIV, Case management approach, Health Systems optimization **Multisectoral interventions** Enabling environment for ALHIV care and treatment, Multisectoral collaboration **Independent management of overall health** Transition to adult care
3.	National Guidelines for Antiretroviral Therapy, sixth Edition	2019	MoHSS, Directorate of Special Programs (DSP)	To address clinical, operational and programmatic aspects of managing the HIV disease and co-morbidities.	Age: 10–19 years (WHO) Further classified as younger adolescents (10-14) and older adolescents (15-19).	**Psychosocial support** Counselling, Mental health, Peer support, HIV disclosure, Caregiver support, Stigma and discrimination **Biomedical interventions** HIV treatment, Viral load monitoring, Medication challenges, Switching ART regimens **Structural interventions** Schooling, Community support systems, Family structural challenges support, Economic/ financial support, Rights-based universal access to HIV services, Protective services, Life skills/Livelihood support **Health Services delivery models** Adolescent and youth-friendly HIV services, Differentiated care service delivery, Case management approach, Integrated Healthcare services package for ALHIV, Health Systems optimization **Multisectoral interventions** Enabling environment for ALHIV care and treatment, Multisectoral collaboration **Independent management of overall health** Transition to adult care, Self-efficacy, treatment literacy
4.	National Guidelines for Antiretroviral Therapy, Pocket Guide	2021	MoHSS, Directorate of Special Programs (DSP)	Updates to the 2019 National Guidelines for Antiretroviral Therapy, sixth Edition of 2019.	Age: 5–19 years age group specifically targeted for HIV disclosure	**Psychosocial support** Mental health, Counselling, Peer support, HIV disclosure, Caregiver support, Stigma and discrimination, Substance abuse **Biomedical interventions** HIV treatment, Viral load monitoring, Medication challenges, Switching ART regimens **Structural interventions** Academic life, Schooling, Community support systems, Family structural challenges support, Economic/ financial support **Health Services delivery models** Differentiated care service delivery, Adolescent and youth-friendly HIV services, Integrated Healthcare services package for ALHIV, Case management approach, Health Systems optimization **Independent management of overall health** Self-efficacy, treatment literacy, Transition to adult care, Participatory approach
5.	Starter Pack and Activity Guide for Teen Clubs	2019	MoHSS I-TECH Namibia Implementing partners and stakeholders Healthcare professionals	Provides standardized guidance to healthcare workers and Teen Club facilitators on how to form a teen club, how to manage it, and include positive activities for meetings, and overall sustaining such support groups	Age: 0–14 (Children), 15–19 (Adolescents) The overall target for teen clubs is 10–19 years.	**Psychosocial support** Mental health, Counselling, Peer support, HIV disclosure, Caregiver support, Stigma and discrimination, Substance abuse **Biomedical interventions** HIV treatment, Viral load monitoring, Medication challenges **Structural interventions** Academic life, Schooling, Community support systems, Family structural challenges support, Economic/ financial support, Life skills/Livelihood support, Protection services **Health Services delivery models** Differentiated care service delivery, Adolescent and youth-friendly HIV services, Integrated Healthcare services package for ALHIV **Independent management of overall health** Self-efficacy, treatment literacy, Transition to adult care, Participatory approach
6.	National Strategic Framework (NSF) for HIV and AIDS Response in Namibia 2017/18 to 2021/22	2017	MoHSS, Directorate of Special Programmes (DSP) Multisectoral & line Ministries Development partners	The NSF provides overall strategic direction for the multisectoral HIV response, describing priority results, key programmes, strategies and target populations. It is the guiding force for the HIV and AIDS response for a 5-year period.	Age: 10–19 years	**Psychosocial support** Counselling, Peer support, Mental health, Caregiver support **Biomedical interventions** HIV treatment **Structural interventions** Academic life, Schooling, Community support systems, Family structural challenges support, Protective services **Health Services delivery models** Differentiated care service delivery, Adolescent and youth-friendly HIV services, Integrated Healthcare services package for ALHIV, Health Systems optimization, **Multisectoral interventions** Multisectoral collaboration
7	Scaling up an evidence-based model of health, happiness and hope for children and adolescents living with HIV across the Africa region: A case study of South-to-South learning (CATS)	Nov 2022	Zvandiri group, Zimbabwe Ministry of Health and Social Services, Namibia	The Zvandiri model offers a comprehensive and evidence-based package of interventions tailored to the holistic needs and challenges of CAYALHIV. Theoretically grounded, multicomponent DSD model	Age: 0–24 years (from childhood, adolescence into adulthood)	**Psychosocial support** Mental health, Counselling, Peer support, HIV disclosure, Caregiver support, Stigma and discrimination, Substance abuse **Biomedical interventions** HIV treatment, Viral load monitoring, Medication challenges, Switching ART regimens **Structural interventions** Academic life, Schooling, Community support systems, Family structural challenges support, Economic/ financial support, Life skills/Livelihood support, Rights-based universal access to HIV services, Protection services **Health Services delivery models** Differentiated care service delivery, Adolescent and youth-friendly HIV services, Integrated Healthcare services package for ALHIV, Case management approach, Health Systems optimization, **Multisectoral interventions** Multisectoral collaboration, Multisectoral leadership, Enabling environment for ALHIV care and treatment **Independent management of overall health** Self-efficacy, treatment literacy, Transition to adult care, Participatory approach

The documents encompassed in this review discuss HIV service provision for a broader demographic inclusive of adolescents (10-19 years). Although they all address issues inclusive of the adolescents’ group, they also target children, adolescents and young adults living with HIV (CAYALHIV) with ages ranging from 0 to 24 years, and adults. The included documents can be classified as guidelines (n = 4),^28,30,31,33^ strategic frameworks/strategy documents (n = 2),^27,29^ and the best practice document (n = 1).^32^ Among the guideline documents, the National Guidelines on Adolescents Living with HIV provides a minimum basic essential package of care and standards for multisectoral interventions to address needs of ALHIV (10–19 years) holistically and improving their quality of life.^33^ The National Guidelines for Antiretroviral Therapy (sixth Edition and Pocket Guide) mainly outline clinical aspects of managing HIV disease and comorbidities in all populations, including the 5–19 years age group.^28,30^ The Starter Pack and Activity Guide for Teen Clubs provides standardized guidance on how to form, manage and sustain teen clubs and activities for the support group for the ALHIV (10–19 years).^31^ On the other hand, the two strategic frameworks/strategy documents include the National Paediatric and Adolescent HIV Care and Treatment Strategy, which focuses more on HIV care and treatment outcomes, including achieving viral suppression for CALHIV (0–19 years), and the NSF which outlines the overall 5-year period strategic direction for the multisectoral HIV and AIDS response in Namibia, describing the target and priority populations, and the key strategies and programs.^27,29^ The best practices document, which describes the scaling up of the Zvandiri model, propagates a comprehensive and evidence-based package of a multicomponent differentiated service delivery (DSD) model, which addresses the holistic needs of CAYALHIV (0–24 years).^32^

Namibian health policy provides strategic directives and guidelines to deliver *psychosocial support for ALHIV*, through counselling, addressing mental health issues, through peer groups and caregiver support. *Biomedical interventions* were prominent in strategic and implementation imperatives, and included viral load monitoring, timely ART regimen switches and medication challenges. *Structural interventions* included components of education, teaching life skills, finances, community and family structures and protective services highlighted as some of the key areas to support ALHIV to ensure they thrive in their communities. *Health services interventions* were emphasized in the policy frameworks and guidelines to foster the implementation of DSD, adolescent-friendly HIV services, integration of healthcare services, and optimization of health systems to adequately monitor treatment outcomes in ALHIV, to enhance sensitivity to ALHIV.

Only five of the seven documents included *multisectoral interventions* and collaboration with a focus on creating an enabling environment for ALHIV care and treatment, while one document emphasized multisectoral leadership involvement. Providing and promoting mechanisms for enhancing *independent management of overall health* among ALHIV appears to be a key component in six of the seven documents. Concepts such as self-efficacy, treatment literacy and participatory approaches are addressed as key components to ensure favourable treatment outcomes for ALHIV, as well as ensuring greater successes in transitioning from paediatric and adolescent care to adult care. As a whole, the policy documents and strategic frameworks are complimentary in addressing the needs of ALHIV from a policy perspective to implementation at ground (health facility and community) level. However, as much as the combined content from individual documents may broadly touch on essential components in management of ALHIV, they fall short in addressing specific needs of unsuppressed adolescents, and providing clear guidance on identifying the root causes of viral non-suppression and steps to manage these cases.

Table 2 outlines the concepts addressed in the content of the guidelines (n = 4),^28,30,31,33^ strategic frameworks/strategy documents (n = 2),^27,29^ and the best practice document (n = 1).^32^ Among the seven documents reviewed, only five intentionally addressed the adolescents (as defined by WHO). The National Guidelines for Antiretroviral Therapy Pocket Guide and the NSF do not explicitly highlight issues related to management of ALHIV.^27,28^ With respect to HIV service delivery, five documents specifically addressed viral non-suppression, in their guidelines and as best practice.^28–30,32,33^ The Starter Pack and Activity Guide for Teen Clubs and the NSF are silent on addressing viral non-suppression in individuals receiving HIV treatment.^27,31^ Six documents outline interventions to improve treatment outcomes in individuals receiving antiretroviral treatment while the NSF does not provide sufficient details.^28–33^ All documents except the National Guidelines for Antiretroviral Therapy Pocket Guide acknowledge the unique challenges for ALHIV and the need to tailor interventions to their unique needs.^27,29–33^ Overall, regarding viral non-suppression (the treatment gap), interventions for improvement in suppression levels considering the lived experiences and challenges of ALHIV (10–19 years) are addressed and outlined in four of the seven documents, the National Guidelines on Adolescents Living with HIV, the Namibia Paediatric and Adolescent HIV Care and Treatment Strategy: 2019–2023, the National Guidelines for Antiretroviral Therapy, Sixth Edition, and the Scaling up an evidence-based model of health, happiness and hope for children and adolescents living with HIV across the Africa region: A case study of South-to-South learning (CATS) documents.^29,30,32,33^

**Table 2.** Analysis of the Key Concepts in Identified Policy and Programmatic Documents.

#	Policy/Document	Adolescents 10–19 years	Viral Non-Suppression	Interventions	Challenges for ALHIV	*Score
1	National Guidelines on Adolescents Living with HIV, second Edition	√	√	√	√	
2	Namibia Paediatric and Adolescent HIV Care and Treatment Strategy: 2019–2023	√	√	√	√	
3	National Guidelines for Antiretroviral Therapy, sixth Edition	√	√	√	√	
4	National Guidelines for Antiretroviral Therapy, Pocket Guide	X	√	√	X	
5	Starter Pack and Activity Guide for Teen Clubs	√	X	√	√	
6	National Strategic Framework (NSF) for HIV and AIDS Response in Namibia 2017/18 to 2021/22	X	X	X	√	
7	Scaling up an evidence-based model of health, happiness and hope for children and adolescents living with HIV across the Africa region: A case study of South-to-South learning (CATS)	√	√	√	√	

*Score card (satisfy all 4 criteria – Adolescents, Viral non-suppression, includes Interventions, and acknowledges Challenges for ALHIV). **Green –** meets all 4 criteria, **Light Green –** meets 3 of the 4 criteria, **Orange –** meets 2 of the 4 criteria, and **Red –** meets only 1 of the 4 criteria.

Six main themes emerged from our thematic analysis of the seven documents included in the review as outlined in Table 3.

**Table 3.** Thematic Analysis of Policy and Programmatic Documents Addressing Management of Adolescents Living with HIV.

Themes	Coded Categories	Codes (Content)
1. **Psychosocial support**	Mental health	Addressing behavioural challenges Healthcare Workers to encourage positive living Linkages to psychiatrists, psychologists, and social workers, for psychosocial support (PSS) PSS to caregivers to avoid burnout Bereavement support for the loss of parents/caregivers PHQ9 for assessment of Depression, Mental health issues Use CETA (Common Elements Treatment Approach) to address mental health issues
	Counselling	Age-appropriate and developmentally appropriate adherence counselling and support Group-based counselling Individually tailored counselling Enhance (Intensified) Adherence Counselling (EAC)/ Treatment failure counselling Treatment literacy on the importance of adherence Adolescent expert clients providing counselling services Addressing scheduling difficulties, use of reminders such as clocks, phones, radios Counselling to improve retention in care/Continuity of Care
	Peer support	Peer-led individual peer-to-peer counselling support Peer mentors and peer educators Peer-led differentiated care models Peer learning and education groups Building strong supportive relationships Trained and mentored peers for YPLHIV, CATS (18-24 years) Young Mentor Mothers (YMMs) and Young Mentor Dads (YMDs) Peer-led HIV services and mental health services
	HIV disclosure	HIV disclosure support for children and adolescents, at home or in healthcare facility Prevent inadvertent disclosure of parents’ status
	Caregiver support (parents, guardians, teachers)	Supporting and capacitating the caregiver Community gatekeepers/other guardians’ engagement Parent-to-child communication skills through guardian or caregiver clubs Guardians and caregivers trained on adherence, disclosure to teachers, and matrons in boarding schools (Caregiver groups and workshops) Evaluate treatment literacy and caregiver/guardian/ parent/ treatment supporter understanding of high viral load Assess caregiver/guardian support Create supportive environments in the community (caregivers, schools, faith and religious leaders, community members)
	Stigma and discrimination	Confidentiality, dealing with stigma and discrimination “Why I take my medicine” booklet for disclosure and addressing stigma, and adherence issues, in local languages Tackle stigma and discrimination in schools, communities, facilities Zero tolerance for stigma and discrimination
	Substance abuse	Substance abuse, refer to CAGE alcohol screening tool
2. **Biomedical interventions**	HIV treatment	Timely treatment initiation despite WHO stage or CD4 count Optimal treatment regimens Address physical health through clinical monitoring Lower ART coverage rates among adolescents Treatment supporters for adherence to ART Forgetfulness, use SMS reminders, DOTS, Pill boxes, medication calendar
	Viral load monitoring	Scale up viral load monitoring among adolescents Monitor ALHIV with unsuppressed VL, evaluate potential virologic failure Targeted VL testing for ALHIV suspected of failing treatment Unstable adolescents (high viral load, social challenges) to be seen more frequently for clinical consultations, care and support
	Medication challenges	Medication side effects Drug tolerability (palatability, nausea, vomiting, diarrhoea) Encourage patients to report adverse events Addressing treatment fatigue, especially among older adolescents Pill burden Consider use of FDC combinations Medicine dosages adjustments accordingly due to stunting or underweight among adolescents
	Switching ART regimens	Transition to more potent regimens, DTG regimens ART regimen switch (changing due to treatment failure) Consider timely switching to second line and third line when indicated
**3. Structural interventions**	Academic life, schooling	School support Review academic performance for school going child/adolescent to assess cognitive development Strengthen school retention programs
	Community support systems	Intergenerational dialogues to address harmful cultural practices and negative social norms Community support Personal and structural aspects of life not in their control Strengthen community systems
	Family structural challenges support	Structural challenges encountered by families/caregivers Strengthening the family Difficult environment the ALHIV is living in, away from the biological parent, child-headed family, extended family
	Economic/financial support	Unlocking other funds that could benefit ALHIV Economic strengthening for ALHIV Financial support for projects Support OVCs with social support and processing of social grants through MGECW Strengthen domestic funding for ALHIV interventions
	Protective services	Strengthen social protection services Violence awareness and prevention A child needing protective services may be directly referred to MGECW
	Life skills/Livelihood support	Discuss future goals and planning, money issues, jobs Skills development, training and tertiary education opportunities Learning sessions on life skills building and entrepreneurship skills Quality of life Youth Empowerment Groups Income generation opportunities Knowledge and skills development, communication skills
	Rights-based universal access to HIV services	Human rights and gender-based needs (Gender Sensitivity and gender equity) Contextualized, non-discriminatory response and services Inclusive care Disability services
4. **Health services delivery**	Differentiated care service delivery	Facility-based group ART refill models Community-based ART refill models, home ART refills, CAGs, Home visits Treatment adherence clubs (Teen Clubs) Synchronize ART refills with teen club meetings WhatsApp groups to support teens with clinical or psychosocial challenges in-between teen club meetings CATS/Zvandiri (Namibia – NATS) Adolescents meeting DSD criteria can have multi-month dispensing (MMD) Addressing health systems issues
	Adolescent and youth-friendly HIV services	Adolescent days Embrace a research agenda targeted at addressing the unique challenges confronting ALHIV Adolescent-friendly treatment services and support for retention Improving the low coverage of adolescent-friendly services
	Integrated Healthcare services package for ALHIV	Comprehensive, integrated, quality care Opportunistic infections management, TB prevention, screening TB/HIV, Nutrition/Malnutrition and other co-morbidities SRHR (family planning, STI management, pregnancy screening, VMMC) PMTCT/eMTCT, PrEP Sexual partners identification, transient relationships Cervical Cancer prevention and screening
	Case management approach	HIV-sensitive case management training for facility-based social workers and nurses Prioritized case management for high viral load cases Pregnant transitioning teenagers and parenting education Case-based surveillance Scheduled Case management system by Case care workers
	Health Systems optimization	Health information systems to track LTFU, monitor viral suppression rates Human Resources for Health, capacity development Supply chain management Community, clinic and digital health services, eHealth, Mobile health Medicine supply chain and laboratory systems Quality improvement initiatives Use TRIOS, treatment supporters, CHWs Long clinic waiting times Update guidelines, policies, and protocols in line with new evidence Strengthen SBCC campaigns to address low health-seeking behaviour, gender norms
5. **Multisectoral interventions**	Multisectoral collaboration	Multisectoral referrals and responses, Partnerships Multisectoral engagement, partnerships with line ministries, partners Leverage multisectoral expertise, reach, knowledge, and resources to address the holistic needs of ALHIV Referrals to Ministry of gender equality and child welfare (MGECW) for <18 with insufficient care, missing appointments, VL failure, poor adherence (after attempts at clinic have failed)
	Enabling environment for ALHIV care and treatment	Enabling environment for management, coordination and implementation of adolescent ART and care Capacity building for health workers, teachers, social workers, parents, police workers
	Multisectoral leadership	Governments multisectoral leadership, line ministries, development partners
6. **Independent management of overall health**	Self-efficacy, treatment literacy	Decision-making, taking responsibility for their own health and disease management Self-sustenance, Self-reliance, Self-care, self-management Independence in taking medication, making appointments, and educational, and financial needs Treatment literacy, self-confidence, problem-solving skills Good, healthy habits Health, Nutrition, physical exercise, happiness, and hope Learning sessions on clinical aspects of HIV, HIV basics Education on HIV prevention (eg, condom use) Foster love and self-acceptance and increase self-esteem
	Transition to adult care	The transition from paediatric to adult care
	Participatory approach	Advocacy led by or through ALHIV Community participation and engagement involving ALHIV Greater and meaningful engagement with ALHIV in planning activities

### Psychosocial Support (PSS)

Identifying and managing **mental health** issues in ALHIV is presented as one of the key focus areas. Guidance is provided for healthcare workers to address behavioural challenges, encouraging positive living, bereavement support, and providing PSS to caregivers of ALHIV. Linkages through appropriate referrals to psychiatrists, psychologists and social workers should be available, including use of appropriate mental health assessment tools. **Counselling**, both through group-based or individually tailored sessions is well articulated. The guidance includes use of age-appropriate and developmentally appropriate adherence counselling, Enhanced Adherence Counselling (EAC), treatment failure counselling, counselling to improve retention in care (continuity of care), including the use of reminders such as clocks, radios or phones. **Peer support** in form of individual peer-to-peer support, peer mentors, peer educators and peer learning in education groups, emerged as a common sub-theme for PSS.

Supporting and capacitating the caregivers, engaging community gatekeepers and treatment supporters for ALHIV is also indicated. The **Caregiver support** outlined includes capacitating parents, guardians, schoolteachers, faith and religious leaders, and community members, to support improvements in treatment outcomes among ALHIV. **HIV disclosure** support for children and adolescents is also highlighted, in the healthcare facilities as well as at home. Confidentiality and having zero tolerance to **stigma and discrimination** in communities, at facilities and in schools are emphasized, as well as addressing issues around **substance abuse**.

### Biomedical Interventions

**HIV treatment** is the cornerstone of biomedical interventions for individuals living with HIV and the documents reviewed highlighted the need for timely initiation of ART regardless of WHO clinical stage or CD4 count, with the use of optimal ART regimens is critical. Guidance on **viral load monitoring** is mostly on scaling up testing of adolescents, targeted monitoring of those with unsuppressed viral loads more regularly and evaluating potential for virologic and treatment failure. Transition to more potent regimens, especially DTG-based regimens, or appropriate **switching of ART regimens** due to treatment failure, in a timely manner, as appropriate, is recommended. Managing **medication challenges** such as side effects, drug tolerability (palatability, nausea, diarrhoea, vomiting), pill burden, treatment fatigue especially among older adolescents, are some of the issues needing attention.

### Structural Interventions

The **academic life** of ALHIV is a key component of the policy and guidelines documents, with schooling support, strengthening school retention programs, and assessing their cognitive development highly recommended. **Community support systems** also feature as key structural support systems to address personal and structural aspects of life. Adolescents may be living in difficult environments, often away from their biological parent, with extended family or in a child-headed family, which all would require appropriate **family structural challenges support**. **Economic and financial support** for income-generating projects, unlocking funding sources to support ALHIV and strengthening domestic funding for ALHIV are some of the highlighted interventions. **Protective services** outlined include violence awareness and prevention and appropriate referrals the MGECW and police services. Consequently, adolescents should have **rights-based universal access to HIV services**, with inclusive care, disability services and to a contextualized, non-discriminatory HIV response and services. The documents also recognise the need to empower the adolescents with **life skills and livelihood support**.

### Health Services Delivery

**Differentiated care service delivery (DSD) models** were quite a common theme in all reviewed documents. At the community level, community-based ART refill models, home visits, and community adherence groups (CAGs) were the key DSD models, while at the facility level there are guidelines for treatment adherence clubs such as teen clubs with synchronized ART refills, facility-based group ART refill models, and mixed models such as the Zvandiri/CATS program. The provision of **adolescent and youth-friendly HIV services** is also recommended. An **integrated healthcare services package** for ALHIV which consists of management of opportunistic infections, malnutrition, other co-morbidities, sexual and reproductive health, Cervical cancer services, as well as managing transient relationships, is outlined. A **case management approach** is recommended, with prioritization of high viral load cases for case management. **Health systems optimization** is also extensively narrated with capacity development for Human Resources for Health (HRH), engaging treatment supporters, and capacitating and supporting community health workers (CHWs). Other systems issues included are developing appropriate governance systems, updated guidelines, policies, and protocols following new evidence.

### Multisectoral Interventions

Multisectoral interventions presented include building an **enabling environment** for the care and treatment of ALHIV through building healthcare worker capacity to address multisector issues, capacity building for teachers, social workers, parents, police workers and other public sector stakeholders. **Multisectoral collaboration** is also outlined with recommendations for multisectoral referrals, response and partnerships with other stakeholders and line ministries, including the role of the MGECW in supporting adolescents <18 years with poor adherence and virologic failure. **Multisectoral leadership** at all levels, including government, line ministries and development partners is also highlighted.

### Independent Management of Overall Health

Along with guidance on the **transition to adult care** from pediatric or adolescent services, concepts such as **self-efficacy and treatment literacy** emerged as precursors to a successful transition. Enhancing decision-making ability, self-sustenance, self-reliance, self-management and adolescents taking responsibility for their own health and treatment are key components towards independent care. Education on HIV prevention and treatment, HIV basics and clinical aspects, enhances treatment literacy, self-confidence, problem-solving skills and encourages adoption of good and healthy habits. Empowered adolescents can then be engaged through **participatory approaches** in advocacy activities led by or through ALHIV, with greater and meaningful engagement of adolescents in planning activities, community participation and overall full involvement in activities that determine their own health.

## Discussion and Conclusions

The health policy and guidelines landscape in Namibia can be described as *inclusive* of adolescents living with HIV, with only two documents being *intentional* to adolescents. Our review identified a package of guidelines (n = 4),^28,30,31,33^ strategic frameworks/strategy documents (n = 2),^27,29^ and the best practice document (n = 1),^32^ that addressed ALHIV needs. The guidelines packages to some extent address viral load suppression among ALHIV and provide guidance on interventions based on the unique experiences of adolescents. However, there may be policy implementation gaps in terms of specifically tackling adolescents with unsuppressed viral loads. The treatment gap among ALHIV may as well be related to gaps in policy and guidelines implementation at all levels, from national to subnational level guidance (facility-level and community-level packages), that result in a persistent 12% gap of viral non-suppression.^18^ Considering the persistent challenges with achieving the last 95% of the UNAIDS targets (viral suppression) among this subpopulation, it may be worthwhile to develop specific operational guidance for this age group.

One of the two frameworks/strategy documents addresses adolescent-specific issues while the overarching national strategy document is silent about adolescent-issues. There are opportunities to highlight the remaining treatment gap for ALHIV and developing specific strategies to address viral non-suppression among adolescents in the next iterations of frameworks/strategy documents. Whereas the best practices document provides a pathway to addressing viral non-suppression, it includes a broader sub-population, and appears more promising in addressing adolescent-specific HIV viral suppression challenges. Admittedly, the dilemma is on having an “Adolescent Health in All Policies” approach, which promotes having sections of the policy documents specifically addressing adolescent health issues, or developing policies and guidelines that are adolescent-specific, intentionally and exclusively addressing adolescent health.^34^

Across the documents reviewed, pertinent issues regarding management of ALHIV are well articulated, albeit in a siloed but complementary fashion. Nevertheless, the seemingly fragmented nature of the information and guidance may need some consolidation into a single strategy that can provide comprehensive guidance to healthcare providers and other stakeholders engaged in managing and supporting ALHIV, especially those challenged with viral non-suppression. The National Guidelines on Adolescents Living with HIV,^33^ provides a resource that could be expanded, with consolidation of strategies, interventions and best practices outlined in the other documents, as well as strengthening issues addressing adolescents with unsuppressed viral loads. Granted, key issues such as psychosocial support, biomedical interventions, structural interventions, health service delivery models, multisectoral interventions and enhancing self-care among ALHIV are outlined across the documents, it is not clear how to expand these interventions in a structured and consistent way to manage cases of adolescents with unsuppressed viral loads.

In addition to the interventions outlined in the reviewed documents, interventions for consideration for comprehensive operational guidance to manage ALHIV with unsuppressed viral loads may include use of Multidisciplinary team approaches to support adolescents failing treatment, age-appropriate clinical and social support that leverage on digital platforms for individual Case Management, supportive school environments for ALHIV, integration of the peer support such as NATS and teen clubs, clear guidance on Enhanced Adherence Counselling issues for ALHIV, Caregiver clubs for unsuppressed adolescents, treatment buddies, PeleBoxes for picking up medication, provision of reminders such as wristwatches and pill boxes, Direct Observed Treatment (DOT) for adolescents with high viral load, Viraemia clinic day, Family clinic for high VL adolescents, Family-based Economic Empowerment, Conditional Economic Incentives and Motivational Interviewing. However, the persisting gaps may as well be due to challenges with implementing the existing policies and guidelines, and lack of clarity on what steps to take when encountering an adolescent challenged with viral suppression. We suggest further research (implementation research) to evaluate the implementation of the policies and guidelines and assess the potential gaps in managing virally unsuppressed ALHIV.

## Study Limitations

The availability of documents online was limited. Most of the documents were physical copies obtained from the Ministry of Health and Social Services and from other experts in the field, and it may be possible that some relevant documents may have been missed. Namibia has not developed it’s own guidelines on the implementation of the Namibia Adolescent Treatment Supporter (NATS), hence we used the Zvandiri Community Adolescent Treatment Supporter (CATS) document.
